# Effect of green tea supplementation on the microbiological, antioxidant, and sensory properties of probiotic milks

**DOI:** 10.1007/s13594-014-0165-6

**Published:** 2014-03-26

**Authors:** Dorota Najgebauer-Lejko

**Affiliations:** Faculty of Food Technology, Department of Animal Product Technology, University of Agriculture in Krakow, ul. Balicka 122, 30-149 Krakow, Poland

**Keywords:** Green tea, Probiotics, Fermented milk, Antioxidant activity, Sensory evaluation

## Abstract

Green tea and its constituents are known for a wide range of health-promoting properties. They may exert antimicrobial action but without altering lactic acid bacteria. The aim of the present study was to estimate the effect of green tea addition on the selected properties of probiotic milks. Bioyogurts (fermented with ABT-1 coculture of *Streptococcus thermophilus*, *Lactobacillus acidophilus* LA-5, *Bifidobacterium animalis* subsp. *lactis* BB-12) and acidophilus milks (fermented with pure *L. acidophilus* LA-5 culture) with addition of 0, 5, 10, or 15% (*v*/*v*) green tea infusion (GTI) were produced and analyzed for the antioxidant capacity by the “diphenyl picrylhydrazyl” (DPPH) and “ferric-reducing antioxidant power” (FRAP) methods, acidity, the count of starter bacteria, and sensory properties at the 1st, 7th, 14th, and 21st day of cold storage. The 15% addition of GTI to the acidophilus milk significantly reduced the lactic acid production during the whole study. The GTI had no impact on the level of *S. thermophilus* and *B. lactis* BB-12 in bioyogurts, and its effect on the count of *L. acidophilus* LA-5 depended on the concentration and probiotic milk type. GTI similarly and in a dose-dependent manner enhanced the antioxidant capacity of both milk types. There were no significant differences between the sensory notes received for bioyogurts, whereas acidophilus milks with tea were less appreciated by the panelists. In conclusion, green tea could be successfully used as a functional additive for selected probiotic milks enhancing their health benefits, but the proper selection of tea additive and starter culture is recommended.

## Introduction

Probiotics are defined as “live microorganisms which, when administered in adequate amounts, confer a health benefit on the host” (FAO/WHO 2006). Fermented dairy products, such as yogurt, are very popular food delivery systems of live probiotic cells. In recent years, due to various therapeutic benefits documented and increased consumer health awareness, the popularity of the functional dairy products containing probiotics has significantly increased. The health benefits linked with the consumption of probiotic microorganisms most commonly used in dairy products, i.e., belonging to *Lactobacillus* and *Bifidobacterium* genera, comprise prevention and relieving effects in various types of diarrhea (infantile, traveler’s, antibiotic-associated), alleviation of gastrointestinal complaints, reduction of lactose intolerance, lowering serum cholesterol level, anticarcinogenic activity, prevention of urogenital infections, reduction of allergic symptoms, stimulation of the immune system, etc. (FAO/WHO 2006; Sanders et al. 2013). It is important that probiotic food products must contain living probiotic strains in an adequate matrix and in sufficient concentration at the time of consumption to reach after ingestion the postulated effect. However, the main problems connected with incorporating probiotic bacteria into fermented milk formulae are their slow growth in milk and loss of viability during storage (El-Dieb et al. 2012). Different strategies can be applied to support the growth of probiotic bacteria in milk, e.g., microencapsulation of probiotic cells, heat shock of the yogurt before addition of probiotics, proper selection of starter cultures, addition of prebiotic substances (e.g., inulin, oligosaccharides), etc. (El-Dieb et al. 2012; Oliveira et al. 2012). Dave and Shah (1997) used ascorbic acid additive as oxygen scavenger to make the environment more conducive, i.e., with reduced oxygen content and redox potential, for these microaerophilic (*Lactobacillus acidophilus*) or strictly anaerobic (*Bifidobacterium* ssp.) microorganisms. Probiotic milks are also often supplemented with other active components with the aim to provide additional functional properties, like plant sterols and stanol esters as well as antioxidative substances (Saxelin 2008).

Tea is the second, next to water, most commonly consumed beverage worldwide. Many studies have been conducted which demonstrated beneficial effects of tea and its constituents on human health. The health claims include reduction of risk of cancer, arteriosclerosis and cardiovascular diseases, neural and obesity problems, diabetes, pulmonary ailments, and diseases of the kidneys and liver, and antibacterial and antiviral effects. The most important bioactive substances responsible for these health-giving properties present in tea are flavonoids (namely, catechins and their derivatives) (Jain et al. 2006). Among all functions of polyphenols, their antioxidant activity is the most frequently studied. Their in vitro action as antioxidants refers to the scavenging activity against reactive oxygen and nitrogen species and the ability to sequester metal ions (Bancirova 2010; Zhu et al. 2006). Polyphenols have been also reported as very potent antimicrobial agents (von Staszewski et al. 2011). The study of Almajano et al. (2008) indicated that tea compounds characterized by the highest antioxidant power are simultaneously the most effective as microbiological inhibitors. Phenolics present in tea and wine are able to modify the intestinal microbiota by inhibiting the growth of pathogenic bacteria and increasing the level of commensal bacteria, including bifidobacteria, which suggests their prebiotic effect (Hara 1997; Lee et al. 2006; Queipo-Ortuño et al. 2012). Catechins from tea and other phenolic compounds have also been shown to inhibit the growth of many food-borne bacteria and fungi in milk with little effect on lactic acid bacteria (Almajano et al. 2008; O’Connell and Fox 2001). The sensitivity of lactic acid bacteria (LAB) and bifidobacteria to the phenolic compounds depends on the bacterial species and strain, as well as chemical structure and concentration of the polyphenols (Tabasco et al. 2011). Some of the resistant strains, e.g., *Lactobacillus plantarum*, *Lactobacillus casei* Shirota, are also able to metabolize these compounds (Lee et al. 2006; Rodríguez et al. 2009).

In addition to their benefits for human health, phenolics also affect sensory properties, i.e., flavor, taste (astringency), and color of the food products (Rodríguez et al. 2009). These facts make tea not only very popular as a beverage, but its extracts are also successfully incorporated into food systems, e.g., ice cream mixes, yogurt, and fruit-flavored milk drinks (O’Connell and Fox 2001).

Previous studies demonstrated that addition of tea extracts to the conventional yogurt, i.e., containing *Streptococcus thermophilus* and *Lactobacillus delbrueckii* subsp. *bulgaricus*, did not affect the viability of starter microorganisms (Jaziri et al. 2009) or the stimulating effect was observed (Najgebauer-Lejko et al. 2011). However, there is lack of information on the effect of tea in fermented milks containing probiotic bacteria. This study was established to estimate the effect of green tea infusion (GTI) supplementation in different concentrations (0, 5, 10, or 15% *v*/*v*) on the antioxidant capacity measured as scavenging activity against DPPH radical and ferric-reducing ability (FRAP) of bioyogurts (milks fermented with the ABT-1 coculture of *S. thermophilus* and two probiotic strains, i.e., *L. acidophilus* LA-5 and *Bifidobacterium animalis* ssp. *lactis* BB-12) and acidophilus milks (fermented with pure *L. acidophilus* LA-5 culture) and to determine whether GTI can affect viability of starter cultures, including probiotic bacteria, during a 3-week cold storage. Moreover, the acidity and sensory quality of the plain and supplemented probiotic milks were evaluated.

## Materials and methods

### Materials

Fresh, raw, cows’ milk (∼20 L) for the production of probiotic milks was obtained from a local milk farm in Dziekanowice (Poland). Chinese leaf green tea (Yunnan Tea Garden Group Shareholding Co., Ltd., Kunming, China) was purchased from the local supermarket. Instant nonfat milk powder was purchased from Dairy Company in Gostyn (Poland). ABT-1 DVS coculture, consisting of *S. thermophilus*, *L. acidophilus* (LA-5), and *B. lactis* (BB-12), and *L. acidophilus* LA-5 monoculture were obtained from Chr. Hansen (Hoersholm, Denmark).

Folin-Ciocalteu’s phenol reagent, Trolox, and gallic acid monohydrate were purchased from Fluka (Buchs, Switzerland; Copenhagen, Denmark; and Madrid, Spain), and 2,2-diphenyl-1-picrylhydrazyl (DPPH) and 2,4,6-tris(2-pyridyl)-*s*-triazine (TPTZ) from Sigma-Aldrich (Steinheim, Germany and Buchs, Switzerland). All other chemicals used were of analytical reagent grade.

### Preparation of tea infusion

Green tea leaves (40 g) were infused in 800 mL of freshly boiled water in a glass-covered beaker for 15 min. The leaves were then removed and the infusion cooled to ambient temperature. To achieve the same content of milk solids in all final products, a boiled-and-cooled-to-ambient-temperature water in the proper amounts was added to milk destined for the nonsupplemented (natural) probiotic milks (bioyogurt and acidophilus milk) as well as for the milks with 5 or 10% (*v*/*v*) of tea infusions.

### Manufacturing of bioyogurts and acidophilus milks

The preparation of milk for probiotic milk products comprised centrifugation (3,500×*g*, 45 °C) to reach 2% fat level, standardization with nonfat milk powder (NMP) to achieve 15% dry matter content in the final products, homogenization (60 °C, 6 MPa), pasteurization (85 °C, 15 min), and cooling to 38 °C. Subsequently, the bulk milk was inoculated with the ABT-1 starter (0.08 g per 1 L of milk) for bioyogurts or LA-5 culture (0.1 g per 1 L of milk) for acidophilus milks. Each treatment was divided into four equal portions; mixed with proper amounts of GTI or/and water to reach 0, 5, 10, or 15% of GTI; and poured into 200-mL sterile glass jars. The incubation proceeded at 37 °C for 10–12 h (the same time for all milks) until firm coagula were formed (pH of 4.6–4.8 for all treatments except for acidophilus milk with 15% of GTI which was higher). Subsequently, fermented milk products were immediately cooled and stored at 4 °C prior to analyses. The samples were subjected to analyses directly after production and after 7, 14, and 21 days of refrigerated storage at ∼4 °C.

### Analyses of tea infusion

Green tea infusion was analyzed for the total level of polyphenolic compounds using the Folin-Ciocalteu method by the procedure described by Rusak et al. (2008). Results expressed as milligrams of gallic acid equivalents (GAE) per liter of infusion were recalculated per 100 g of the respective milk. Additionally, the content of flavan-3-ols, i.e., (−)-epicatechin (EC), (−)-epigallocatechin (EGC), (−)-epigallocatechin gallate (EGCG), and (−)-epicatechin gallate (ECG), was determined by means of high-performance liquid chromatography (HPLC). The catechins were identified using L-7000 LaChrom liquid chromatograph with UV/Vis detector (Merck-Hitachi, Tokyo, Japan) equipped with a reversed phase (ODS) column (Thermo Scientific, USA; 25 mm × 0.4 cm × 5 μm). The sample preparation and exact analysis followed the procedure described by Socha et al. (2013). The determination of ferric-reducing antioxidant power (FRAP) and scavenging rate of 2,2-diphenyl-1-picrylhydrazyl (DPPH) radical followed the procedures described in the previous work of Najgebauer-Lejko et al. (2011). The FRAP was expressed as millimoles Fe^2+^ equivalents (E) per liter, and the DPPH antiradical activity as antiradical power (ARP) calculated relative to the ARP of Trolox in millimoles of Trolox equivalents (TE) per kilogram sample.

### Analyses of fermented milks

In all samples, pH was measured using an Elmetron (Zabrze, Poland) CP-411 pH meter. Titratable acidity expressed as percentage of lactic acid was determined according to the Soxhlet-Henkel method (Polish Standard, PN-A-86061:2002).

The level of *S. thermophilus* in bioyogurts was assessed using M17 agar (Biocorp, Warszawa, Poland) under aerobic conditions. For enumeration of *L. acidophilus*, MRS-maltose agar was prepared from the same ingredients and procedure as MRS agar (ISO 7889:^2003^) but by replacing glucose with an equal amount of maltose and adjusting pH to 6.4. The Petri dishes for lactobacilli enumeration in bioyogurts and acidophilus milks were incubated respectively under aerobic conditions or in 2.5-L anaerobic jars using Anaerocult® C sachets (Merck, Darmstadt, Germany). The count of bifidobacteria was evaluated using nalidixic acid, neomycin sulfate, lithium chloride, and paromomycin sulfate (NNLP)-MRS agar (MRS supplied by Biocorp, Warszawa, Poland) in anaerobic incubators of our own construction filled with CO_2_. All cultures were incubated at 37 °C for 2 (streptococci) or 3 (lactobacilli, bifidobacteria) days.

The evaluation of the antioxidant capacity was also performed using DPPH and FRAP methods by the same procedures as for the tea infusion. The sensory evaluation was performed according to the PN-ISO 6658 standard (1998) using a 5-point scale (from 5—excellent to 1—very bad) by a trained panel of five judges. The fermented milk samples were presented to panelists in identical plastic cups labeled with random numbers and in random order. The following properties were assessed: color, taste, odor, consistency, and general appearance. The overall preference was calculated taking into account the proper indexes of importance for each quality attribute (0.10, 0.35, 0.15, 0.25, 0.15, respectively).

### Statistical analysis

All experiments were performed in three series and in duplicate and results expressed as mean ± standard error. Estimation of the effect of tea addition and time of storage was conducted using ANOVA, and where the significant effect was found, the significance of differences between the means was determined on the basis of Duncan’s test at the significance level of *P* ≤ 0.05. The statistical analysis was performed using Statistica 8.0 software (StatSoft Inc., Tulsa, OK, USA).

## Results

### Composition and acidity of fermented milks

The bioyogurts and acidophilus milks produced in this study contained on average 15.2% of dry matter (dm), 1.7% of fat, and 4.8% of protein as well as 0, 0.07, 0.15, or 0.22 g of tea dry matter per 100 g of fermented milk, respectively, for each level of supplementation (data not shown). The addition of GTI to the examined probiotic milks resulted in the enrichment of their composition with tea polyphenols in the amount of 57.9, 115.8, and 173.8 mg GAE per 100 g of fermented milk (for 5, 10, or 15% of tea supplementation, respectively), as determined by the Folin-Ciocalteu method. HPLC analysis revealed that the green tea infusion used for milk supplementation contained 81.25 ± 0.23 (−)-epicatechin (EC), 148.47 ± 0.24 (−)-epicatechin gallate (ECG), 147.36 ± 4.78 (−)-epigallocatechin (EGC), and 242.86 ± 1.90 (−)-epigallocatechin gallate (EGCG) (mg⋅100 mL^−1^ of tea infusion).

Directly after production, it could be observed that the pH of the bioyogurts and acidophilus milks was slightly higher for higher levels of green tea supplementation, which was accompanied by the lower values of titratable acidity (Table 1). This phenomenon was much more pronounced in the case of milk products fermented solely with *L. acidophilus* LA-5 showing the noticeable difference of 0.51 units and 0.42% lactic acid, respectively, for initial pH and titratable acidity noted between plain acidophilus milk (NA) and that with 15% of green tea infusion (GTA-15%). The significant difference in acidity of NA and GTA-15% was also observed after storage. Generally, the pH values decreased and the level of lactic acid slightly increased throughout the storage, although observed pH reduction was statistically significant (*P* ≤ 0.05) only for acidophilus milk with 15% of green tea. The drop in pH value observed for this treatment was above twice that evaluated for the other milks fermented with *L. acidophilus* LA-5 monoculture.

**Table 1:** Changes of the pH, titratable acidity, and sensory notes of bioyogurts and acidophilus milks during cold storage (*n* = 6)

Sample	NY	GTY-5%	GTY-10%	GTY-15%	NA	GTA-5%	GTA-10%	GTA-15%	SE
Day	pH								
1	4.54 a	4.61 a	4.70 ab	4.78 ab	4.51 a	4.62 a	4.69 ab	5.02 bA	0.05
7	4.54 ab	4.52 ab	4.58 ab	4.70 ab	4.38 a	4.48 ab	4.59 ab	4.83 b	0.05
14	4.50 ab	4.48 ab	4.60 ab	4.64 ab	4.32 a	4.48 ab	4.60 ab	4.74 b	0.04
21	4.48 a	4.50 a	4.50 a	4.65 a	4.34 a	4.45 a	4.59 a	4.64 aB	0.03
Day	Titratable acidity (% lactic acid)								
1	1.07 ab	0.91 ab	0.87 ab	0.70 a	1.16 b	1.07 ab	0.95 ab	0.74 a	0.05
7	1.13 ab	1.12 ab	1.08 ab	0.75 a	1.25 b	1.22 b	1.09 ab	0.94 ab	0.05
14	1.12 ab	1.14 ab	0.96 ab	0.81 a	1.31 b	1.08 ab	1.00 ab	0.92 ab	0.05
21	1.13 ac	1.08 ac	0.94 ac	0.81 a	1.31 c	1.24 bc	1.11 ac	0.90 ab	0.04
Day	Sensory evaluation (scores)								
1	4.77 a	4.72 a	4.78 a	4.70 a	4.53 a	4.19 a	4.20 a	4.17 a	0.07
7	4.78 c	4.69 bc	4.79 c	4.62 bc	4.04 ab	3.81 a	3.71 a	3.52 a	0.12
14	4.86 d	4.82 d	4.79 d	4.71 cd	4.43 bcd	4.07 ac	3.83 ab	3.67 a	0.10
21	4.66 b	4.60 b	4.48 b	4.43 b	4.20 ab	3.99 ab	3.68 a	4.14 ab	0.10

Means within each row not sharing the same lowercase letter are statistically different (*P* ≤ 0.05); different capital letters given in columns denote the statistical difference (*P* ≤ 0.05) between means for a given feature

*NY* natural (plain) bioyogurt; *GTY-5%*, *GTY-10%*, and *GTY-15%*—bioyogurts with 5, 10, and 15% (*v*/*v*) of green tea infusion, respectively; *NA* natural (plain) acidophilus milk; *GTA-5%*, *GTA-10%*, and *GTA-15%*—acidophilus milks with 5, 10, and 15% of green tea infusion, respectively; *SE* standard error of the mean

### Antioxidant capacity of probiotic milks as affected by green tea supplementation

**Fig. 1:**
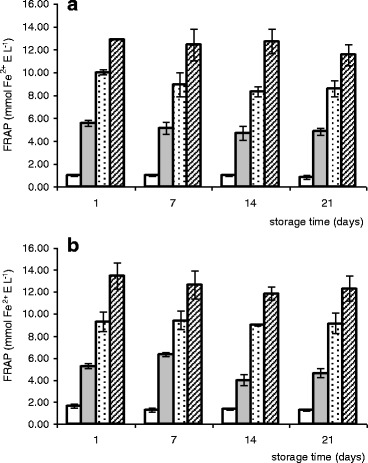
Changes of the ferric-reducing antioxidant power (FRAP) values of bioyogurts (**a**) and acidophilus milks (**b**) during refrigerated storage (□ natural bioyogurt/acidophilus milk;  bioyogurt/acidophilus milk with 5% of green tea infusion;  bioyogurt/acidophilus milk with 10% of green tea infusion;  bioyogurt/acidophilus milk with 15% of green tea infusion; means ± SE, *n* = 6)

**Fig. 2:**
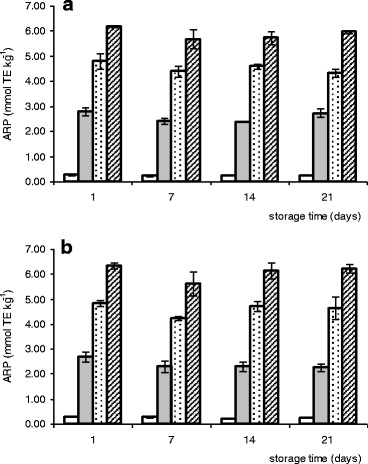
Changes of the antiradical power (ARP) values of bioyogurts (**a**) and acidophilus milks (**b**) during refrigerated storage (□ natural bioyogurt/acidophilus milk;  bioyogurt/acidophilus milk with 5% of green tea infusion;  bioyogurt/acidophilus milk with 10% of green tea infusion;  bioyogurt/acidophilus milk with 15% of green tea infusion; means ± SE, *n* = 6)

Two methods were employed for measuring antioxidant activity of fermented milks, i.e., DPPH assay, which measures the scavenging activity of the DPPH radical by antioxidant substances present in the examined sample, and FRAP, which allows to estimate the ability to reduce prooxidant metal ions. The results of FRAP evaluation expressed as millimoles Fe^2+^ E per liter and DPPH analysis given as ARP in millimoles of Trolox equivalents (TE) per kilogram sample are presented in Figs. 1 and 2, respectively.

In the present study, ARP values evaluated for the plain bioyogurt (NY) and acidophilus milk (NA) fluctuated in the range of 0.21–0.28 mmol TE kg^−1^ with no significant differences between these two types of fermented milks (*P* > 0.05). The FRAP values were slightly higher for NA (1.25–1.61 mmol Fe^2+^ E L^−1^) than those for NY (0.89–1.05 mmol Fe^2+^ E L^−1^), but also in this case, the differences were insignificant. Green tea infusion was characterized by noticeably higher antioxidant capacity with 41.60 ± 0.73 mmol TE kg^−1^ and 72.76 ± 6.74 mmol Fe^2+^ E L^−1^ average ARP and FRAP values, respectively (data not shown).

The strong antioxidative properties of green tea resulted in significantly higher ARP (9–29-fold) and FRAP (3–13-fold) values of all supplemented fermented milks when compared with the natural treatments. The higher level of tea additive was applied, the higher radical-scavenging ability and ferric-reducing activity was observed, with statistically significant differences between the respective results (*P* ≤ 0.05). There were no differences between bioyogurts and acidophilus milks with the same level of supplementation. For all products, the FRAP and ARP values determined after 3 weeks of storage were lower than the initial by 2–22% and 2–16% (respectively), but the rate of this decrease was statistically insignificant.

### Effect of green tea addition on the viability of the starter bacteria

**Fig. 3:**
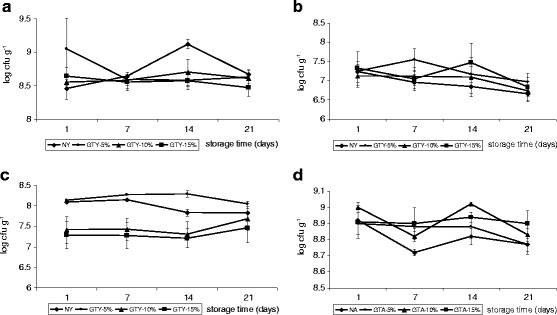
Viability of starter bacteria in probiotic milks with or without green tea additive during refrigerated storage: **a** *Streptococcus thermophilus* count in bioyogurts, **b** *Bifidobacterium animalis* ssp. *lactis* count in bioyogurts, **c** *Lactobacillus acidophilus* count in bioyogurts, and **d** *Lactobacillus acidophilus* count in acidophilus milks. *NY* natural bioyogurt; *GTY-5%*, *GTY-10%*, and *GTY-15%*—bioyogurt with 5, 10, and 15% of green tea infusion, respectively; *NA* natural acidophilus milk; *GTA-5%*, *GTA-10%*, and *GTA-15%*—acidophilus milk with 5, 10, and 15% of green tea infusion, respectively (*bars* denote standard error of the mean)

The effect of green tea addition to the bioyogurts and acidophilus milks on the number of starter microorganisms is shown in Fig. 3a–d. As regards probiotic strains, lactobacilli were present in the number of 7.21–8.29 log cfu g^−1^ (bioyogurts) or 8.72–9.02 log cfu g^−1^ (acidophilus milks) and the count of bifidobacteria in bioyogurts reached the level of 6.66–7.54 log cfu g^−1^. The levels of all starter bacteria in both types of probiotic milks and in all treatments remained practically unchanged after 3 weeks of refrigerated storage (no significant differences at *P* ≤ 0.05).

*S. thermophilus* with the number of 8.47–9.12 log cfu g^−1^ was the prevailing species in bioyogurts constituting from 69 to 95% (depending on storage duration and level of tea supplementation) of all starter microbiota. As revealed by the analysis of variance (data not shown), the green tea additive did not affect the count of streptococci in bioyogurts. Some fluctuations observed in their number in the plain bioyogurt (NY) and the one containing 5% of green tea infusion (GTY-5%; Fig. 3a) during storage were insignificant. In bioyogurts with higher levels of tea incorporation, the concentration of cocci was stable within the studied period.

Statistical analysis revealed that the number of bifidobacteria was unaffected by the bioyogurt type, but it is worth to emphasize that the count of these probiotic bacteria remained at a higher level for a longer period of time in fermented milks enriched with green tea (decrease below 7 log cfu g^−1^ after 3 weeks in bioyogurts with green tea vs. 1 week for NY). The concentration of *B. animalis* subsp. *lactis* BB-12 in overall population of starter microorganisms determined in the obtained probiotic yogurts ranged between 1 and 5% for nonsupplemented and fortified with 5 and 10% of GTI bioyogurts, whereas in GTY-15%, the average bifidobacteria share was ∼10%. According to the manufacturer’s stated colony-forming unit counts, the lactobacilli to bifidobacteria to streprococci ratio at inoculation should have been 2:2:1. Thus, major changes occurred in the strain ratio during fermentation.

The effect of GTI incorporation into the bioyogurt formulation on the viability of *L. acidophilus* LA-5 was dose-dependent. The higher the dosage of GTI, the lower number of these bacteria evaluated in fermented milk. The 5% green tea supplementation resulted in significantly higher number of lactobacilli (by almost one log cycle) than higher doses of GTI. In relation to the plain bioyogurt, the difference was negligible.

Acidophilus milks contained higher amounts of lactobacilli than bioyogurts (8.72–9.02 vs. 7.21–8.29 log cfu g^−1^). In the case of milk fermented with *L. acidophilus* LA-5 in monoculture, the green tea infusion at 10 and 15% influenced the higher level of bacteria than 0 or 5% treatment. The milks which contained 5 and 15% of GTI were very stable as regards lactobacilli content within the study period, whereas in NA and GTA-10%, some fluctuations in the number of these bacteria were observed.

### Effect of tea addition on the sensory properties of probiotic milks

The incorporation of tea extracts into the bioyogurt had no significant influence on the notes received in the sensory evaluation (Table 1). The notes fluctuated in the range of 4.43–4.86 and were slightly lower at the end of the experiment, although the storage time was an insignificant factor as regards this feature. Acidophilus milks were worse appreciated by the panelists than bioyogurts (3.52–4.53), which was connected with less acceptable flavor as well as consistency and general appearance mainly due to visible whey separation (data not shown). In this case, fermented milks supplemented with tea received lower notes than plain acidophilus milk and with one exception (GTA-15% at the 21st day), the higher tea additive was related to lower notes.

## Discussion

The main phenolic compounds present in green tea responsible for its high antioxidant potential are catechins: (−)-epigallocatechin gallate (EGCG), (−)-epigallocatechin (EGC), (−)-epicatechin gallate (ECG), and (−)-epicatechin (EC) as well as gallic acid (Cabrera et al. 2003). Research data usually focus on EGCG as the very powerful tea antioxidant. EGCG was the most abundant flavan-3-ol compound detected in green tea infusion in the present study. Milk also possesses some antioxidant activity resulting from the presence of such components as bioactive peptides derived from both caseins and whey proteins, lactoferrin, urate, ascorbate, α-tocopherol, β-carotene, coenzyme Q10, and enzymatic systems (superoxide dismutase, catalase, and glutathione peroxidase), which is a property that in fermented milk can be further improved as starter microorganisms also possess some antioxidant potential (Chen et al. 2003; Kullisaar et al. 2002). Much more higher DPPH radical-scavenging and ferric-reducing abilities of green tea resulted in certain enhancement of this property in fermented milks with every increase of tea supplementation.

The results obtained for acidity measurement in this study, which suggest that GTI slowed down the acidity development during fermentation and storage, are opposite to those obtained for the conventional yogurt, where 5–15% green tea additive (regardless of the concentration) resulted in significantly lower pH values and simultaneously higher *L. delbrueckii* subsp. *bulgaricus* count when compared with the plain yogurt (Najgebauer-Lejko et al. 2011). On the other hand, Jaziri et al. (2009) demonstrated that green tea extract had no effect on the lactic acid concentration and bacteria survival in yogurts, while data from Gaudreau et al. (2013) are at least partially in agreement with the detrimental effect of higher concentrations of tea extracts on the growth of lactobacilli in bioyogurts as observed in the present study. In ABT yogurt culture used in the present study, *L. bulgaricus* is excluded, which significantly reduces the pH decrease during production and storage as this species is a better lactic acid producer than *L. acidophilus* and *Bifidobacterium* sp. (Lourens-Hattingh and Viljoen 2001; Shihata and Shah 2002).

To exert the beneficial health effects, the amount of probiotic bacteria in the food product should be adequately high, i.e., 10^6^–10^8^ cfu mL^−1^ throughout the entire shelf life (Ghoddusi and Hassan 2011). The level of *L. acidophilus* and *B. animalis* ssp. *lactis* evaluated during the whole storage period (21 days) in this study met this criterion. Enhanced viability of bifidobacteria in tea-supplemented yogurts during the first 2 weeks of refrigerated storage may be connected with higher pH values of bioyogurts containing higher amounts (10 and 15%) of green tea (pH ≥ 4.6) as growth of most strains of bifidobacteria is retarded at pH values below 4.6 (Lourens-Hattingh and Viljoen 2001).

In the study conducted on milk fermented with traditional yogurt culture, the positive effect of green tea infusion (the same tea and preparation procedure as in the present study) at the concentration of 10 and 15% was observed, whereas 5% supplementation negatively affected the population of streptococci when compared with natural yogurt (Najgebauer-Lejko et al. 2011). On the contrary, Jaziri et al. (2009) reported that green or black tea had no effect on the lactic acid bacteria in yogurt. This suggests that the effect of tea supplementation on the growth and survival of selected starter microorganisms in milk systems, among other factors (type of tea and its composition, procedure of tea yogurt preparation, etc.), depends also on the composition of starter microbiota used for fermentation.

In the present study, LA-5 probiotic strain of *L. acidophilus* was used for milk fermentation in the monoculture and in the coculture with *S. thermophilus* and *B. lactis* BB-12. The viability of lactobacilli was influenced by both the green tea concentration and type of fermented milk product. These findings are in concordance with the observations of Tabasco et al. (2011) that the sensitivity of lactic acid bacteria (LAB) and bifidobacteria to the phenolic compounds depends on the bacterial species and strain as well as chemical structure and concentration of the polyphenols. In their study, the growth of tested *L. acidophilus* LA-5 strain was negatively affected by the addition of flavan-3-ol-enriched grape seed extract. On the contrary, Hervert-Hernández et al. (2009) reported the stimulatory effect of grape phenolic extract and some of its pure components (tannic acid, catechins) on the growth of probiotic lactobacilli (*L. acidophilus* CET 903). Almajano et al. (2008) showed the inhibitory effect of extracts of different teas against food-borne pathogens, e.g., *Bacillus cereus*, *Micrococcus luteus*, and *Pseudomonas aeruginosa*, whereas *L. acidophilus* exhibited exceptional resistance to all extracts studied. The different response of *L. acidophilus* in its pure culture and coculture to the different concentrations of green tea extract applied needs to be considered taking into account the interactions between starter microorganisms in ABT yogurt. The effect of the accompanying species used to ferment milk on the survival of *L. acidophilus* and *Bifidobacterium* spp. was reported by Lourens-Hattingh and Viljoen (2001). Moreover, as some species of the lactobacilli were demonstrated to have the ability to metabolize phenolic compounds (Lee et al. 2006; Rodríguez et al. 2009), the effect of the potential phenolic metabolites cannot be excluded.

Saxelin and coauthors (2003) stated that despite the health benefits, fermentation of milk with pure probiotic strain may result in a product with texture and taste that does not meet the consumer approval; therefore, the common practice is to use probiotic strains together with standard starter cultures, e.g., yogurt. This often takes place in the case of acidophilus milk as *L. acidophilus* is a homofermentative bacterium that gives plain acid flavor, which is frequently perceived as too sour with lack of real aromatic flavor (Lengkey and Adriani 2009). The results of our study suggest that the main taste sensations found in green tea, such as astringency and bitterness (Chaturvedula and Prakash 2011), did not compose well with the taste of acidophilus milk. The possible phenolic metabolites (not studied herein) may also influence sensory characteristic of the product.

## Conclusion

The incorporation of GTI in a dose-dependent manner increased antiradical and ferric-reducing power of probiotic milks with no significant differences between the bioyogurts and acidophilus milks with the same level of supplementation. Bioyogurts with different levels of green tea infusion did not vary as regards the average counts of *S. thermophilus* and *B. animalis* ssp. *lactis* BB-12, but GTI at all applied concentrations maintained the viability of bifidobacteria at the level above 7 log cfu g^−1^ for an additional 2 weeks compared to the plain bioyogurt. Green tea at the concentration of 5% was more beneficial for the viability of *L. acidophilus* in bioyogurt, whereas 10 and 15% positively affected LA-5 growth in monoculture.

In summary, the results of the present study suggest that green tea can be successfully employed as a functional supplement for probiotic milks, adding extra value to the known health benefits of probiotics, but the proper amount of tea additive and cultures for milk fermentation need to be carefully chosen.
